# Non-tuberculous Mycobacteria isolated from Pulmonary samples in sub-Saharan Africa - A Systematic Review and Meta Analyses

**DOI:** 10.1038/s41598-017-12175-z

**Published:** 2017-09-20

**Authors:** Catherine Okoi, Suzanne T. B. Anderson, Martin Antonio, Sarah N. Mulwa, Florian Gehre, Ifedayo M. O. Adetifa

**Affiliations:** 1 Vaccines and Immunity Theme, Medical Research Council Unit, Fajara, The Gambia; 2 Clinical Services Department, Medical Research Council Unit, Fajara, The Gambia; 3 Microbiology and Infection Unit, Warwick Medical School, University of Warwick, Coventry, United Kingdom; 4 Faculty of Infectious and Tropical Diseases, London School of Hygiene & Tropical Medicine, London, United Kingdom; 5 Disease Control and Elimination Theme, Medical Research Council Unit The Gambia, Fajara, The Gambia; 6 Institute of Tropical Medicine, Antwerp, Belgium; 7 Department of Infectious Diseases Epidemiology, London School of Hygiene and Tropical Medicine, London, United Kingdom; 8 Epidemiology and Demography Department, KEMRI-Wellcome Trust Research Programme, Kilifi, Kenya; 9 College of Medicine University of Lagos, Lagos, Nigeria

## Abstract

Pulmonary non-tuberculous mycobacterial (NTM) disease epidemiology in sub-Saharan Africa is not as well described as for pulmonary tuberculosis. Earlier reviews of global NTM epidemiology only included subject-level data from one sub-Saharan Africa country. We systematically reviewed the literature and searched PubMed, Embase, Popline, OVID and Africa Wide Information for articles on prevalence and clinical relevance of NTM detection in pulmonary samples in sub-Saharan Africa. We applied the American Thoracic Society/Infectious Disease Society of America criteria to differentiate between colonisation and disease. Only 37 articles from 373 citations met our inclusion criteria. The prevalence of pulmonary NTM colonization was 7.5% (95% CI: 7.2%–7.8%), and 75.0% (2325 of 3096) occurred in males, 16.5% (512 of 3096) in those previously treated for tuberculosis and *Mycobacterium avium* complex predominated (27.7% [95% CI: 27.2–28.9%]). In seven eligible studies, 27.9% (266 of 952) of participants had pulmonary NTM disease and *M*. *kansasii* with a prevalence of 69.2% [95% CI: 63.2–74.7%] was the most common cause of pulmonary NTM disease. NTM species were unidentifiable in 29.2% [2,623 of 8,980] of isolates. In conclusion, pulmonary NTM disease is a neglected and emerging public health disease and enhanced surveillance is required.

## Introduction

The epidemiology of pulmonary disease caused by *Mycobacterium tuberculosis* complex (MTBC) - *M*. *tuberculosis*, *M*. *bovis*, *M*. *africanum*, *M*. *canetti*, *M*. *microti*, *M*. *pinnipedii* and *M*. *caprae* - is better known than for NTM^1^. NTM is a designation used for a large number of potentially pathogenic and non-pathogenic environmental mycobacterial species other than MTBC and *Mycobacterium leprae*.

Worldwide, pulmonary infections caused by NTM are gaining increased attention, in part, because of their increasing recognition and isolation in clinical settings, for example with better known NTM pathogens such as *M*. *avium subsp paratuberculosis*, *M*. *marinum*, etc.^2,3^. Although NTM were identified soon after Koch’s identification of *M*. *tuberculosis* as the cause of active tuberculosis in 1882, it was not until the 1950s that NTM were recognized to cause human pulmonary disease. Given their ubiquitous presence in the environment, it is important to distinguish colonization from active disease following isolation of NTM from pulmonary samples. In response to this challenge, the ATS/IDSA introduced stringent diagnostic criteria with clinical, radiological and microbiological components for diagnosis of pulmonary NTM disease^2^.

The clinical and molecular epidemiology of prevalent NTM in low and middle-income countries, also endemic for pulmonary tuberculosis, is less known because pulmonary and other disease manifestations caused by NTM pose a diagnostic challenge to microbiologists and clinicians^2,4^. In contrast to pulmonary tuberculosis, it is not possible to readily identify pulmonary NTM disease with the usual combination of basic mycobacteriology, clinical history, radiologic imaging and the tuberculin skin test, where applicable. The culture and molecular biology identification techniques required for NTM diagnosis are not cost effective for routine clinical practice in resource-poor health systems where priority is currently given to expanding access to diagnosis and treatment for pulmonary tuberculosis^5,6^. The distribution of NTM species isolated from pulmonary samples differs significantly by geographic region. However, most of these data are from the developed world and sub-Saharan Africa is under represented^7,8^. Although there are now emerging NTM disease data from Asia and parts of Africa, significant knowledge gaps still exist especially in sub-Saharan Africa where nine of the world’s 22 high burden tuberculosis countries are found^8–11^. Therefore, fears that inconclusive diagnosis based on smear microscopy or clinical symptoms and/or radiological findings could lead to misdiagnosis of pulmonary tuberculosis and/or inappropriate management of pulmonary NTM cases are valid. As it is especially to difficult to differentiate between NTM colonisation and NTM disease the American Thoracic Society/Infectious Disease Society of America (ATS/IDSA) defined a set of clinical and microbiological criteria to diagnose pulmonary NTM disease (Table 1).

**Table 1:** Summary of the American Thoracic Society/Infectious Disease Society of America diagnostic criteria for pulmonary non-tuberculous mycobacterial infection/disease^2^.

**Clinical**
1. Pulmonary symptoms, nodular or cavitary opacities on chest radiograph, or a high-resolution computed tomographic scan that shows multifocal bronchiectasis with multiple small nodules.
**And**
2. Appropriate exclusion of other diagnoses.
**Microbiologic**
1. Positive culture results from at least two separate expectorated sputum samples (If the results from the initial sputum samples are non-diagnostic, consider repeat sputum acid-fast bacillus (AFB) smears and cultures).
**OR**
2. Positive culture results from at least one bronchial wash or lavage.
**OR**
3. Transbronchial or other lung biopsy with mycobacterial histopathological features (granulomatous inflammation or AFB) and positive culture for NTM or biopsy showing mycobacterial histopathological features (granulomatous inflammation or AFB) and one or more sputum or bronchial washings that are culture positive for NTM.
4. Expert consultation should be obtained when NTM are recovered that are either infrequently encountered or that usually represent environmental contamination.
5. Patients who are suspected of having NTM lung disease but who do not meet the diagnostic criteria should be followed until the diagnosis is firmly established or excluded.

The objectives of this review are to consolidate existing data on NTM colonisation and disease (according to ATS/ISDA criteria) in sub-Saharan Africa, review the existing gaps in our knowledge of pulmonary NTM and identify future research priorities.

## Methods

### Literature Search and Selection Criteria

This review was conducted in accordance with PRISMA guidelines^12^. The overall aim of this review was to determine the prevalence of NTM in apparently healthy and diseased individuals in sub-Saharan Africa. We defined sub-Saharan Africa as all of Africa except Northern Africa.

### Search strategy

We searched PubMed, EMBASE, POPLINE, OVID and Africa Wide Information electronic databases for publications about pulmonary NTM in sub-Saharan Africa published from January 1, 1940 to October 1, 2016 using the following search terms and strategy: ((((((“nontuberculous mycobacteria”[MeSH Terms] AND “africa south of the sahara”[MeSH Terms]) OR “mycobacterium infections, nontuberculous”[MeSH Terms]) AND “africa south of the sahara”[MeSH Terms]) OR “mycobacterium infections, nontuberculous”[MeSH Terms]) AND “africa south of the sahara”[MeSH Terms]) OR ((“lung”[MeSH Terms] OR “lung”[All Fields] OR “pulmonary”[All Fields]) AND “nontuberculous mycobacteria”[MeSH Terms])) AND “africa south of the sahara”[MeSH Terms] AND ((“1940/01/01”[PDAT]: “2016/10/01”[PDAT]) AND “humans”[MeSH Terms]).

### Selection process and data abstraction

**Figure 1:**
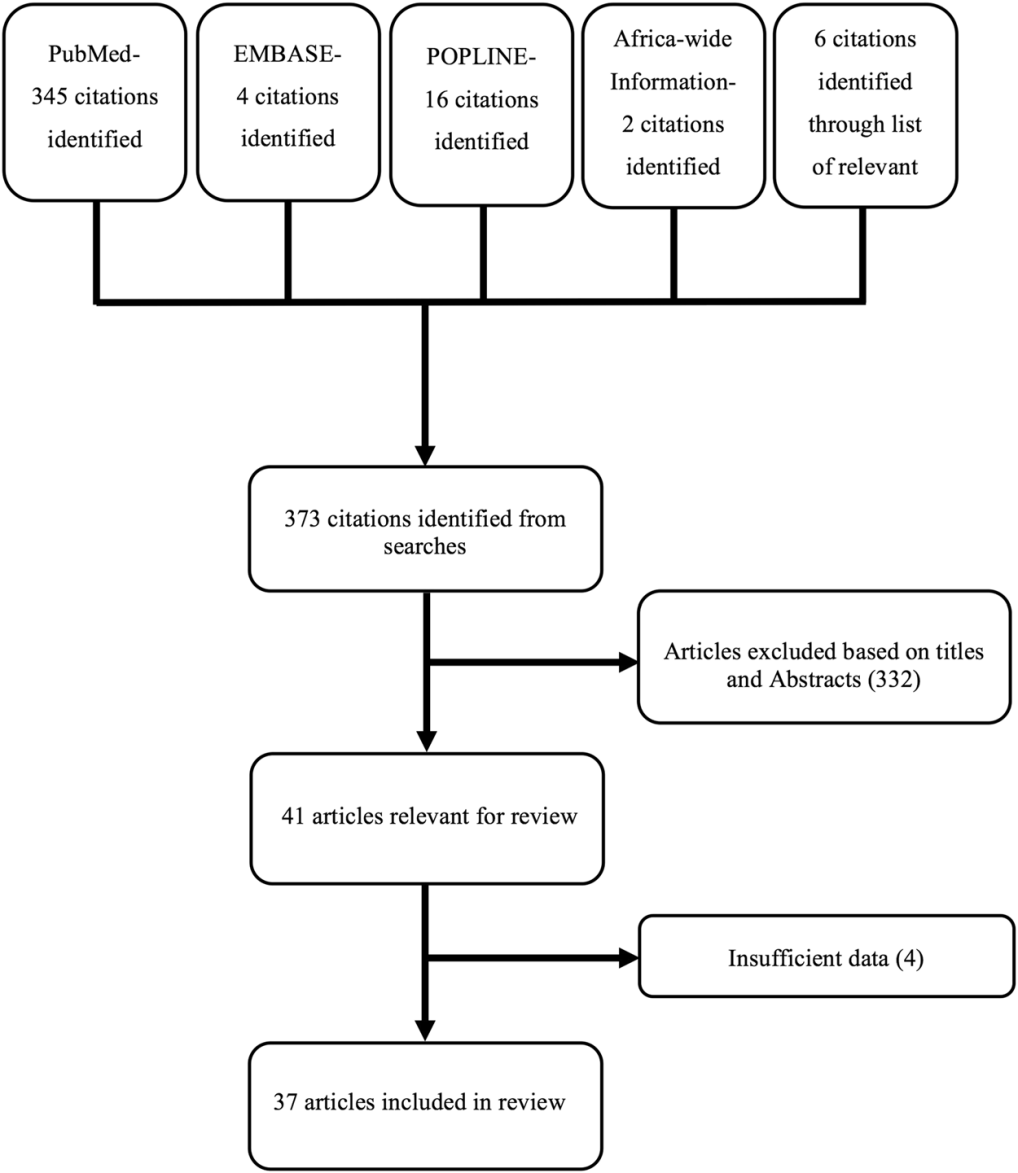
Flow chart of literature search and article selection criteria.

We found 373 citations from our database searches (see Fig. 1). The titles and abstracts of all the articles were screened and full-text copies of those deemed relevant obtained. In addition, the reference sections of all the retrieved articles were screened to identify other eligible citations. Only articles reporting on pulmonary samples were included. For all relevant articles, we extracted the following data using a data extraction sheet: research setting, study period, population tested and numbers, NTM species isolated, method for NTM identification, prevalence of pulmonary NTM isolation/disease, HIV co- infection rate and risk factor(s) for NTM acquisition.

### Data analysis

In estimating country-level and overall prevalence of NTM in sub-Saharan Africa, a pooled estimate was computed based on a simple meta-analysis of the reported prevalences. Each study was weighted according to its sample size and the exact binomial used to derive the 95% confidence intervals (95% CI). We checked all retrieved articles for application of the ATS/IDSA diagnostic criteria (Table 1) for clinically relevant pulmonary NTM and recorded the proportion of patients meeting these criteria and NTM species responsible.

All extracted data were stored in Microsoft® Excel® (Microsoft Corporation, Redmond, Washington, United States) and analysis carried out in STATA™ version 12.1 (College Station, Texas, United States).

## Results

### Description of included studies

There were only 37 relevant articles on the epidemiology of pulmonary NTM in sub-Saharan Africa as shown in Table 2. These were from studies in western (Nigeria, Mali and Ghana), southern (Zambia and South Africa [RSA]) and eastern (Kenya, Uganda, Tanzania and Ethiopia) Africa^5,6,8,10,13–44^. Eleven articles were from Nigeria^5,13,15–21,45,46^, one from Mali^22^, one from Ghana^23^, six from Zambia^6,10,24–27^, two from Kenya^28,29^, two from Uganda^30,31^, three from Tanzania^32–34^, three from Ethiopia^35–37^ and eight from South Africa^8,38–40,43,44^.

**Table 2:** Overview of studies on pulmonary non-tuberculous mycobacteria in sub-Saharan Africa.

Country	Study period	Reference	Age in years	Sample size	Sputum cultures		Most isolated NTM	Method of NTM identification	Overall prevalence of NTM isolation (%)	Pulmonary NTM patients with HIV coinfection (%)	ATS/IDSA applied/numbers meeting criteria	Risk factors for pulmonary NTM
					MTBC	NTM						
Ethiopia	2010	Mathewos *et al*.^36^	NA	263 presumptive TB cases	110	7	NTM not classified	Immunochro-matography assay (Capilia TAUNS method)	2.7	NA	No	NA
Ethiopia	2011	Workalemahu *et al*.^37^	1–15	121 presumptive TB cases	15	10	*M*. *fortuitum M*. *parascrofulaceum M*. *triviale*	Molecular (Sequencing of *16S rRNA* gene)	8.3	NA	NA	NA
Ethiopia	2008–09	Gumi *et al*.^35^	NA	260 presumptive TB cases	157	7	*M*. *flavescens*	Molecular (Sequencing of *16S rRNA* gene)	2.7	NA	No	NA
Ghana	2013–14	Bjerrum *et al*.^23^	≥18	473 HIV infected adults	60	38	*M*. *avium* complex *M*. *chelonae M*. *simiae M*. *fortuitum*	Molecular (sequencing of *16S rRNA* gene)	8.0	All HIV infected	No	HIV infection and age
Kenya	2007–09	Nyamogoba *et al*.^28^	≥0	872 presumptive TB cases	346	15	*M*. *fortuitum M*. *peregrinum*	Molecular (Genotype CM/As assay)	1.7	46.7	No	Previous TB HIV infection
Kenya	2014–15	Limo *et al*.^29^	≥0	210 retreatment cases	121	89	*M*. *intracellulare M*. *abscessus M*. *fortuitum*	Molecular (Genotype CM/As assay)	42.4	25.8	No	Previous TB infection
Mali	2004–09	Miaga *et al*.^22^	18–73	142 presumptive TB cases enrolled	113	17	*M*. *avium M*. *palustre M*. *fortuitum*	Molecular (sequencing of *16S rRNA* gene)	12.0	17.6	Yes; 11	Previous TB
Nigeria	2010–11	Olutayo *et al*.^13^		319 presumptive TB cases	122	26	NA	Molecular (Genotype CM/AS assay)	8.2	46.2	No	HIV infection, age
Nigeria	2008–09	Cadmus *et al*.^46^	NA	23 presumptive cases	11	9	*M*. *avium* complex	Molecular (Sequencing of *16S rRNA* gene)	39.1	NA	No	NA
Nigeria	2010–11	Gambo *et al*.^15^	NA	952 presumptive TB cases	254	65	NTM not classified	Molecular (Genotype CM/AS assay)	6.8	40.0	No	HIV infection, TB
Nigeria	2010–11	Gambo *et al*.^5^	18	1603 TB presumptive TB cases	375	69	*M*. *intracellulare M*. *abscessus M*. *fortuitum M*. *gordonae*	Molecular (Genotype CM/AS assay)	4.3	40.0	No	HIV infection, TB
Nigeria	2008–09	Asuquo *et al*.^16^	10–70	137 presumptive TB cases	81	4	*M*. *fortuitum M*. *avium species M*. *abscessus*	Molecular (Genotype CM/AS assay)	2.9	50.0	No	HIV infection
Nigeria	1983	Idigbe *et al*.^17^	NA	668 presumptive TB cases	NA	NA	*M*. *avium M*. *kansasii M*. *fortuitum*	Conventional biochemical methods	11.0	NA	NA	NA
Nigeria	1982-93	Idigbe *et al*.^18^	NA	NA	NA	NA	*M*. *avium M*. *kansasii M*. *xenopi M*. *fortuitum*	Conventional biochemical methods	NA	NA	No	NA
Nigeria	NA	Mawak *et al*.^45^	≥14	329 presumptive cases	50	15	*M*. *avium M*. *kansasii M*. *fortuitum*	Conventional biochemical methods	4.6	NA	No	NA
Nigeria	2007–09	Daniel *et al*.^19^	25–80	102 TB patients (41 new s + and 61 s + retreatment cases)	70	7	*M*. *fortuitum M*. *intracellulare M*. *chelonae*	Conventional biochemical methods	6.9	15.0	No	Previous TB
Nigeria	NA	Allana *et al*.^20^	NA	NA	NA	NA	*M*. *avium M*. *kansasii M*. *fortuitum*	Conventional biochemical methods	NA	NA	NA	NA
Nigeria	1963	Beer *et al*.^21^	≥1	NA	2682	149	Runyon 111 and 1 V organisms	Conventional biochemical methods	6.0	NA	No	Previous TB
South Africa	2006–07	Clare *et al*.^38^	Median age–44	2496 presumptive TB cases	421	232	*M*. *kansasii M*. *gordonae*	Conventional biochemical methods	9.3	31.9	No	HIV infection
South Africa	1996–97	Corbett *et al*.^39^	NA	TB presumptive cases	NA	118	*M*. *kansasii M*. *fortuitum M*. *scrofulaceum*	Conventional biochemical methods	NA	34.0	Yes; 32	Previous TB, silicosis
South Africa	1993–96	Corbett *et al*.^40^	≥18	594 mine workers	NA	406 NTM	*M*. *kansasii M*. *fortuitum M*. *avium* complex	Conventional biochemical methods	68.4	13.1	Yes; 206	HIV infection, silicosis
South Africa	1993–96	Corbett *et al*.^39^	≥18	243 NTM infected suspects	92	243	*M*. *kansasii M*. *fortuitum M*. *intracellulare*	Conventional biochemical methods	100	NA	No	Previous TB, silicosis
South Africa	1993–96	Corbett *et al*.^40^	≥18	406 gold miners	NA	261 NTM patients	*M*. *kansasii M*. *scrofulaceum*	Conventional biochemical methods	64.3	NA	No	Previous TB, HIV infection
South Africa	2001–05	Hartherill *et al*.^43^	18 (13–23) months	1732 presumptive TB cases	94	109	*M*. *intracellulare M*. *gastri M*. *avium*	Molecular (RFPCR of 65 KD *hsp*gene)	6.3	4.2	Yes; 8	Previous TB
South Africa	2009	Sookan *et al*.^44^	NA	200 NTM suspects	NA	133 NTM patients	*M*. *avium* complex. *M*. *RGM M*. *gordonae*	Molecular (Genotype CM/AS assay)	66.5	NA	No	NA
South Africa	2008	Hoefsloot *et al*.^8^	NA	NA	NA	5646 NTM patients	MAC *M*. *kansasii M*. *scrofulaceum M*. *gordonae*	Molecular (Genotype CM/AS assay, AccuProbe assays, *hsp 65* PCR–restriction enzyme analysis, Inno–Lipa Mycobacteria and biochemical methods	NA	NA	NA	NA
Tanzania	2012–13	Hoza *et al*.^33^	40 7–88	372 presumptive TB cases	85	36	*M*. *gordonae M*. *interjectum M*. *avium* complex *M*. *scrofulaceum*	Molecular (Genotype CM/AS assay)	9.7	33	No	HIV infection and age
Tanzania	2011	Haraka *et al*.^34^	35	1 HIV negative patient with prior TB	NA	1	*M*. *intracellulare*	Molecular (Genotype CM/AS assay)	100	100	Yes;1	Previous TB
Tanzania	2001–13	Katale *et al*.^32^	NA	472 presumptive TB cases	NA	12	*M*. *chelonae M*. *abscessus M*. *spaghni*	Molecular (Sequencing of *16S rRNA* gene)	2.5	NA	No	NA
Uganda	2009	Asimwe *et al*.^30^	12–18	2200 (710 infants and 1490 adolescents presumptive TB cases)	8	95	*M*. *fortuitum M*. *szulgai M*. *gordonae*	Molecular (Genotype CM/As assay)	4.3	NA	No	NA
Uganda	2012–13	Bainomugisa *et al*.^31^	NA	241 presumptive TB cases	95	14	*M*. *avium M*. *kansasii*	Molecular (Polymerase Chain Reaction of 16S rDNA using the Light cycler)	5.8	NA	No	NA
Zambia	2009–12	Mwikuma *et al*.^25^	NA	91 NTM suspected isolates	NA	54	*M*. *intracellulare M*. *lentiflavum*. *M*. *avium*	Molecular (Genotype CM/As assay)	59.3	NA	No	NA
Zambia	NA	Kapta *et al*.^24^	≥1	6123 presumptive TB cases enrolled	265	923	NTM not identified	Immunochromatography assay (Capilia TAUNS method)	15.1	5.8	No	TB and HIV infection
Zambia	2001	Buijtels *et al*.^26^	≥15	167 chronically ill patients	74	93	*M*. *intracellulare M*. *lentiflavum M*. *chelonae*	Molecular (Sequencing of *16S rRNA* gene)	55.6	79.0	Yes; 7	Previous TB HIV infection
Zambia	2001	Buijtels *et al*.^10^	≥25	4 presumptive TB cases	NA	4	*M*. *lentiflavum M*. *goodie*	Molecular (Sequencing of *16S rRNA* gene)	100.0	33.0	No	HIV infection, damaged lungs
Zambia	2011-12	Malama *et al*.^27^	NA	100 presumptive TB cases	46	9	*M*. *intracellulare M*. *abscessus M*. *chimera*	Molecular (Sequencing of *16S rRNA* gene)	9.0	NA	NA	NA
Zambia	2002–03	Buijtels *et al*.^6^	≥15	565 (180 chronically ill patients and 385 healthy controls)	205	93	*M*. *intracellulare M*. *lentiflavum*. *M*. *avium*	Molecular (Sequencing of *16S rRNA* gene)	16.5	45.6	Yes; 1	Previous TB HIV infection, and use of tap water

NA = Data not available in article.

Where methods of identification were reported, molecular techniques (n = 26) were the most frequently used to identify NTM species, followed by conventional biochemical testing identification tools (n = 9) and immunochromatographic assays (n = 2). The molecular diagnostic methods used were Restriction Fragment Polymerase Chain Reaction (RFPCR) of the 65KD *hsp* gene, Genotype CM/AS assay (Hain Life science, Nehren, Germany), and *16S rRNA* gene sequencing analysis in one, eleven and fourteen studies respectively. Identification methods also varied over time and a dramatic rise in the use of molecular methods was observed in the period 2000-2016. Biochemical and phenotypic tools were the only methods used for NTM identification before 2000. Despite this transition in identification methods used over time, there was no major change in diversity of NTM species isolated in the period before and after the year 2000 as shown in Table 3.

**Table 3:** Non–tuberculous mycobacteria species isolated from sub-Saharan Africa, 1965–2016^‡^.

Non-tuberculous mycobacteria species	Prior 2010 Biochemical identification methods	After 2010 Molecular identification methods	Previously associated with disease
*M*. *intracellulare*	Y	Y	Y
*M*. *avium*	Y	Y	Y
*M*. *kansasii*	Y	Y	Y
*M*. *chelonae*	Y	Y	Y
*M*. *abscessus*	Y	Y	Y
*M*. *fortuitum*	Y	Y	Y
*M*. *scrofulaceum*	Y	Y	Y
*M*. *lentiflavum*	Y	Y	Y
*M*. *interjectum*	Y	Y	Y
*M*. *peregrinum*	Y	Y	N
*M*. *gordonae*	Y	Y	N
*M*. *xenopi*	Y	Y	Y
*M*. *malmoense*	Y	Y	Y
*M*. *moriokaense*	Y	Y	N
*M*. *kumamotonense*	N	Y	N
*M*. *kubicae*	Y	Y	N
*M*. *gordonae*	Y	Y	N
*M*. *simiae*	Y	Y	Y
*M*. *palustre*	Y	Y	Y
*M*. *indicus pranii*	N	Y	N
*M*. *elephantis*	N	Y	N
*M*. *flavascens*	Y	Y	N
*M*. *bouchedurhonense*	N	Y	N
*M*. *chimera*	N	Y	Y
*M*. *europaeum*	N	Y	N
*M*. *neoaurum*	N	Y	N
*M*. *asiaticum*	Y	Y	N
*M*. *nonchromogenicum*	N	Y	N
*M*. *gastri*	Y	Y	N
*M*. *nebraskense*	Y	Y	N
*M*. *confluentis*	Y	Y	N
*M*. *porcinum*	Y	Y	Y
*M*. *terrae*	Y	Y	N
*M*. *seoulense*	Y	Y	N
*M*. *engbackii*	Y	Y	N
*M*. *parascrofulaceum*	Y	Y	N
*M*. *triviale*	Y	Y	N
*M*. *scrofulaceum*	Y	Y	Y
*M*. *szulgai*	Y	Y	Y
*M*. *heckeshornense*	Y	Y	N
*M*. *poriferae*	Y	Y	N
*M*. *spaghni*	Y	Y	N
*M*. *goodie*	Y	Y	N
*M*. *aurum*	Y	Y	N
*M*. *conspicum*	Y	Y	N
*M*. *mucogenicum*	Y	Y	N
*M*. *rhodesia*	Y	Y	N
*M*. *gilvum*	Y	Y	N
*M*. *genevanse*	N	Y	N
*M*. *intermidium*	N	Y	N
*M*. *fortuitum 11/M*. *magaritense*	N	Y	Y

Y = isolated N = not isolated. ^‡^Data retrieved from refs^5,6,8,10,16–40,43–46^.

## Synthesis of Results

### Epidemiology of Non-tuberculous Mycobacteria

The overall prevalence of NTM in pulmonary samples in sub-Saharan Africa derived from all 37 papers reviewed was 7.5% (95% CI: 7.2–7.8%). The median age of participants was 35 (Interquartile range, IQR 16–80) years based on 17 of 37 studies with age data. The majority (2325 [75.0%] of 3096) of subjects with NTM were males. Patients in 12 of 37 studies (32.4%) had a previous history of pulmonary tuberculosis and 15 (40.5%) were co-infected with HIV.

**Figure 2:**
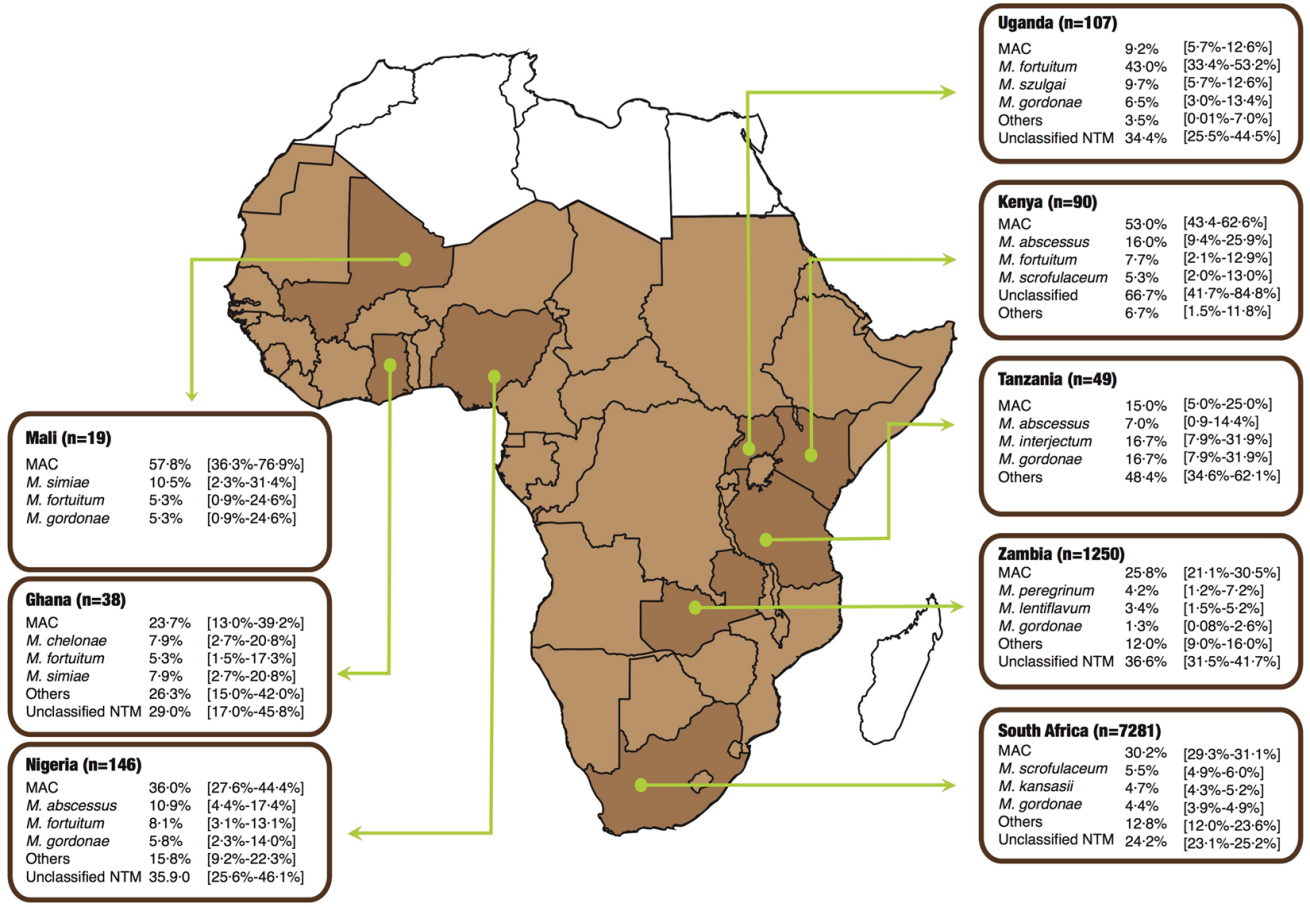
The distribution of the top four non-tuberculous mycobacteria species identified from pulmonary samples in Mali (HIV 1.4%), Ghana (HIV prevalence 1.3), Nigeria (HIV 3.1%), Uganda (HIV 7.1%), Kenya (HIV 5.9%), Tanzania (HIV 4.7%), Zambia (HIV 12.9%), and Republic of South Arica (HIV 19.2%), without considering clinical relevance. Data compiled from refs^5,6,8,10,13,15–17,19–33,35–46^. HIV prevalence compiled from ref.^47^.

MAC species accounted for 28.0% (95% CI: 27.2–28.9%) of all NTM isolated and was the most frequently encountered NTM found in pulmonary samples in 19 of 37 studies. The prevalence of MAC ranged from 15.0% (95% CI: 5.05–25.0%) in Tanzania to 57.8% (95% CI: 36.3–76.9%) in Mali as shown in Fig. 2 (along with country HIV prevalence in the legend^47^). There was regional variability in the distribution of NTM for example; 76.4% (95% CI: 74.8–77.9%) i.e. 2,355 of 3,084 MAC isolates from South Africa were *M*. *intracellulare*, while all MAC isolates from Mali were *M*. *avium*. Similarly, while *M*. *kansasii* was the third most isolated NTM in sub-Saharan Africa overall (4.7% [95% CI: 4.3–5.1%]), it was the top NTM in five (62.5%) of eight studies in South Africa.

Other slow growing mycobacteria isolates, though less prevalent than MAC, were *M*. *scrofulaceum* 7.0% (95% CI: 6.4–7.5%) and *M*. *gordonae* 3.8% (95% CI: 3.4–4.3%). The rapidly growing mycobacteria i.e. *M*. *fortuitum*, *M*. *chelonae*, and *M*. *abscessus* accounted for just 1.2% (95% CI: 1.0–1.4%) of all NTM isolates from sub-Saharan Africa. Rapidly growing mycobacteria were reported predominantly from eastern African countries with *M*. *fortuitum* (43.0% [95% CI: 34.4–53.2%]) and *M*. *abscessus* (16.0% [95% CI: 9.4–25.9%]) as the top and second ranked NTM isolates from Uganda and Kenya respectively.

Among the 70.8% (6357 of 8980) fully speciated isolates in this review, there were 0.9% (56) *M*. *lentiflavum*, 0.9% (55) *M*. *malmoense*, 0.7% (43) *M*. *xenopi*, 0.4% (24) *M*. *gastri*, 0.3% (16) *M*. *szulgai*, 0.2% (15) *M*. *flavescens*, and 0.2% (11) *M*. *interjectum*. Unfortunately, 29.2% (95% CI: 28.1–30.1%) i.e. 2,623 of all 8,980 NTM isolates were not identified to species level.

### Epidemiology of Pulmonary Non-tuberculous Mycobacterial Disease

One particular challenge in studying NTM infection is the difficulty in differentiating between NTM colonisation of patients (due to the mere presence of the bacteria in the environment) and actual pulmonary disease. Therefore the American Thoracic Society/Infectious Disease Society of America (ATS/IDSA) defined a combination of stringent clinical and microbiological criteria to conclusively determine pulmonary disease (see Table 1). To evaluate the geographical distribution of disease-causing NTM only, we excluded 30 articles that only reported on the detection of NTM without applying ATS/IDSA criteria and therefore could not show evidence of pulmonary disease. Only seven (19.0%) of the 37 articles were ATS/ISDA compliant and could be investigated in respect to the epidemiology of clinically relevant NTM^6,22,26,34,39,40,43^. Although these studies had 3,319 participants, only 962 (28.9%) had sufficient information to apply the ATS/IDSA criteria and of these, 266 (27.7%) met the definition of pulmonary NTM disease. *M*. *kansasii*, isolated in 184 (69.2%) of 266 cases, was the most predominant cause of confirmed pulmonary NTM disease, followed by *M*. *scrofulaceum* (13.9%), MAC (13.5%), *M*. *lentiflavum* (1.9%), *M*. *simiae* (0.8%), *M*. *palustre* (0.4%) and *M*. *abscessus* (0.4%).

**Figure 3:**
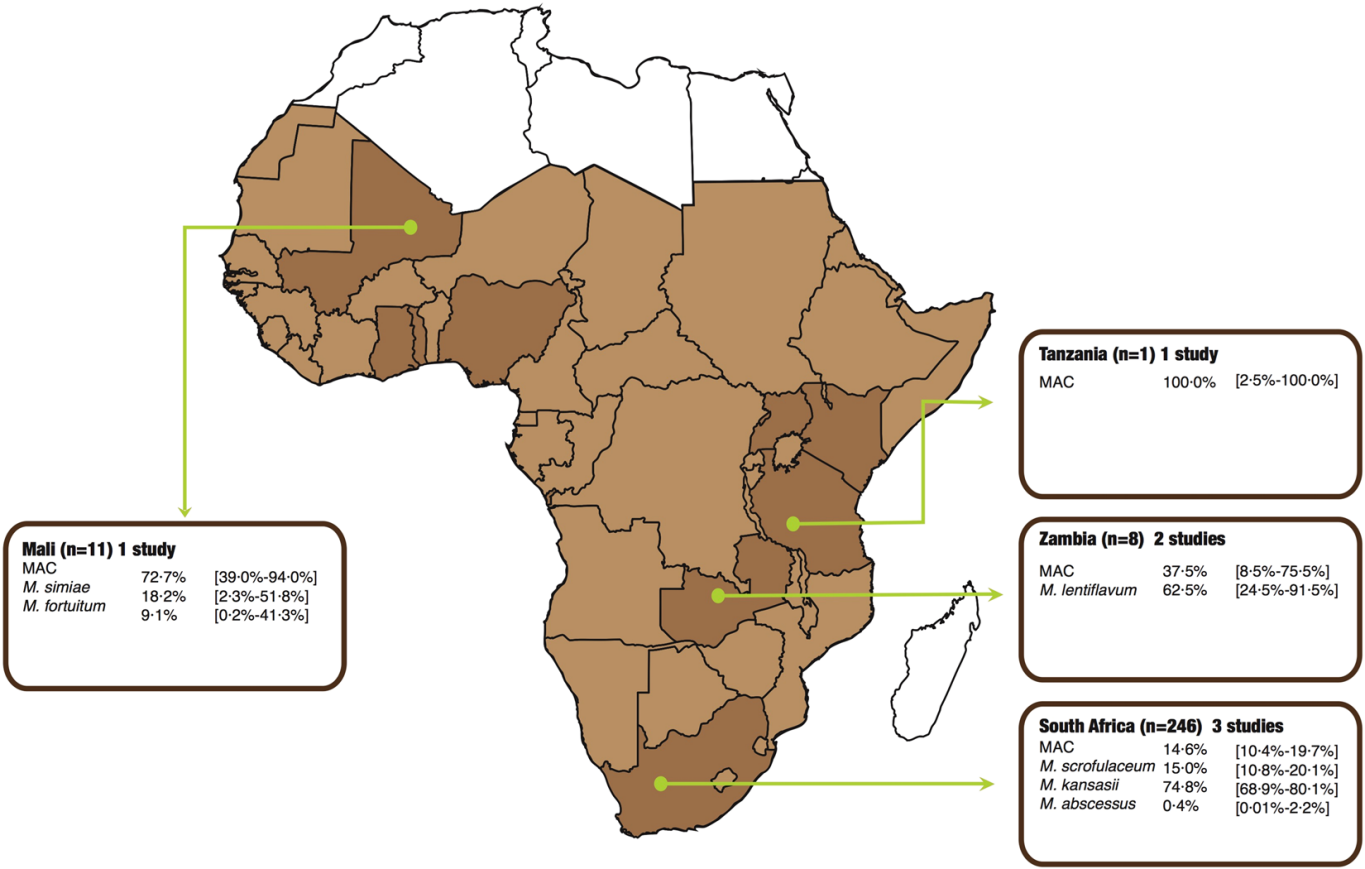
Non-tuberculous mycobacteria species causing pulmonary disease (based on ATS/ISDA criteria) found in respiratory specimens in sub-Saharan Africa. Data compiled from refs^6,22,26,34,39,40,43^.

Figure 3 shows the distribution of NTM species causing pulmonary NTM disease in sub-Saharan Africa by country. The studies investigating the clinical relevance of NTM isolates varied substantially in design, participant characteristics and background HIV prevalence (see Table 2). They ranged from a Zambian study that evaluated the clinical relevance of NTM isolated from 180 chronically ill patients and 385 healthy controls and found only 1.1% of isolates were clinically relevant^6^, to a Malian study in patients with primary and chronic pulmonary tuberculosis where 57.9% of isolated NTM were clinically relevant^22^.

### Clinical and Radiological Signs, and Associated Morbidities

Of 3096 participants with NTM isolates, 80.7% (2498) and 87.5% (2,709) had clinical and radiological information respectively^5,6,15,16,21,22,24–26,29,34,38–40,43,45^. Clinical characteristics for NTM subjects closely mimicked those of pulmonary tuberculosis, as summarized in Table 4. There were radiological abnormalities in 79.0% (2141) of 2709 subjects, while 21.0% (568) had normal chest radiographs. Of the 512 with prior lung disease, 87.1% (446) had a history of tuberculosis and 12.9% (66) had bronchiectasis. In those with concurrent conditions, 50.2% (442) of 880 were coinfected with HIV, 28.2% (248) reported gastrointestinal diseases and 8.6% (76) complained of body weakness. Other characteristics are shown on Table 4.

**Table 4:** Clinical and radiographic characteristics for patients with pulmonary non-tuberculous mycobacteria infections in sub-Saharan Africa, 1965–2016 (N = 3096).

Characteristic	Numbers (%)
**Clinical signs n = 2**, **498**	
Cough ≥ 2 weeks	950 (38.0%)
Chest pain	684 (27.3%)
Significant weight loss	546 (21.9%)
Fever ≥ 2	455 (18.2%)
Night sweats	211 (8.4%)
Haemoptysis	27 (1.1%)
Dyspnoea	19 (0.8%)
**Previous lung disease n = 512**	
Bronchiectasis	66 (12.9%)
Tuberculosis	446 (87.1%)
**Radiographic findings n = 2709**	
Abnormal, suggestive of TB	1009 (37.2%)
No pathological changes	568 (20.9%)
Tuberculosis	446 (16.5%)
Nodules	203 (7.5%)
Fibrosis	140 (5.2%)
Cavitation	127 (4.7%)
Prior focal radiological scarring	107 (4.0%)
Bronchiectasis	66 (2.4%)
Abnormal, not consistent with TB	24 (0.9%)
Milliary TB	19 (0.7%)
**Concurrent conditions n = 880**	
HIV infection	442 (50.2%)
Gastrointestinal disease	248 (28.2%)
Weakness	76 (8.6%)
Lymph node enlargement	52 (6.0%)
Splenomegaly	21 (2.4%)
Diabetes mellitus	22 (2.5%)
Hepatomegaly	19 (2.2%)

## Discussion

We provide an overview of the epidemiology and geographical distribution of NTM species isolated from pulmonary samples in sub-Saharan Africa. To our knowledge, this is the first comprehensive review of pulmonary NTM in this part of the world. Similar to reviews by other authors, our findings suggest diversity in prevalent NTM species, geographical variation in NTM distribution and their clinical relevance across the sub-continent^48^.

The global collection of NTM isolated from pulmonary samples reported by Hoefsloot *et al*.^8^ in 2008 included isolates from only one sub-Saharan Africa country, South Africa. The update in 2013 by Kendall *et al*. did not improve significantly on the earlier review with respect to additional African NTM isolates^1^. Despite the relative scarcity of local data, it is important to highlight that this review is the first to include NTM data for nine countries in sub Saharan Africa.

Overall, we found a predominance of MAC from pulmonary samples in countries of Western, Eastern and Southern Africa. *M*. *scrofulaceum* and *M*. *kansasii* were predominant in Southern Africa and the rapidly growing mycobacteria (*M*. *abscessus*, *M*. *fortuitum* and *M*. *chelonae*) in Eastern Africa. These findings are consistent with the predominance of MAC in the epidemiology of NTM in North America^1,2,49^, Europe^50^, Australia^51^ and East Asia^14^. The relative preponderance of the two members of the MAC family also varied by region with *M*. *intracellulare* predominating in South Africa while all MAC isolates from Mali were *M*. *avium*. However, the South African study had a much bigger sample size compared to the Malian study. While MAC was the most frequently implicated NTM in colonisation, *M*. *kansasii* was the most common in pulmonary NTM disease. The dominance of *M*. *kansasii* as well as the presence of *M*. *scrofulaceum* in South Africa was speculated to be linked to mining activities and significant urbanisation in the country, resulting in a socio-economically disadvantaged population^7,52,53^, working in the mines, frequently suffering from silicosis, while living in poor, overcrowded environments (also see Table 2). When the South Africa pulmonary NTM data is excluded, MAC is the major cause of pulmonary NTM disease as reported in North America, Europe, Australia and Asia^1^. Because relatively few studies in this review applied the ATS/IDSA criteria for confirmation of pulmonary NTM disease, it is difficult to reach conclusions regarding the dominant NTM species causing pulmonary disease in sub-Saharan Africa.

The reason for the observed geographical variation in NTM populations across Africa is still unknown, and could be due to environmental factors associated with the differing geographical country locations. Unfortunately included studies were not designed to investigate sub-regional geographical variation and did not systematically collect environmental data. Ideally future studies on NTM in Africa could address this issue.

In contrast to observations from other parts of the world, especially in Europe, where *M*. *malmoense* and *M*. *xenopi* are well known for causing pulmonary NTM disease^1,54,55^, these NTM were not represented in the limited number of studies reviewed here. *M*. *xenopi* was rare in sub-Saharan Africa, which is not unexpected considering its association with hot water delivery systems that are less developed in sub-Saharan Africa compared to industrialised countries^2,56^.

Pulmonary NTM was commonly associated with a history of previous pulmonary tuberculosis in sub-Saharan Africa compared to Europe and North America. This is not surprising given the high incidence of MTBC disease in sub-Saharan Africa^57,58^. Pulmonary tuberculosis is associated with significant sequelae that have not been adequately studied in sub-Saharan Africa. The associated structural lung damage, chronic pulmonary obstructive disease and infections most likely favour colonization by NTM and other pathogens^59^. It is also likely that the increasing isolation of NTM has come from investigation of patients with chronic pulmonary disease including those complicating previous pulmonary tuberculosis^6,22^. In light of this, the clinical, radiological and microbiologic criteria of the ATS\IDSA is important for distinguishing colonization from pulmonary NTM, particularly in sub-Saharan Africa that is endemic for MTBC^60^.

Many rarely isolated NTM were also identified in presumptive tuberculosis patients, for example *M*. *genavense*, *M*. *gilvum*, *M*. *intermedium*, *M*. *poriferae*, *M*. *spaghni*, *M*. *interjectum*, *M*. *peregrinum*, *M*. *moriokaense*, *M*. *kumamotonense and M*. *kubicae*. Although some of these species have also been isolated in other parts of the world from pulmonary samples in patients with chronic bronchitis, pulmonary tuberculosis, sub-acute pneumonia and healed tuberculosis^61,62^, it is currently unclear what role they play in the aetiology of pulmonary disease in Africa.

The HIV-driven increase in the risk of tuberculosis disease in sub-Saharan Africa has been well described and for NTM, MAC is a particularly well described opportunistic infection in patients with AIDS. We found almost half of all cases of confirmed pulmonary NTM were also HIV co-infected. This suggests the possibility of HIV attributable pulmonary NTM beyond the now familiar disseminated MAC disease often seen in persons with AIDS.

Persons with pulmonary NTM infection in sub-Saharan Africa are younger than observed in North America, Europe and Australia where increasing age (≥50 years), structural lung damage, immunosuppressive chemotherapy for cancer, autoimmune and rheumatoid conditions are the most frequently reported risk factors for this disease^1,2,59,63^. Given the younger age and higher burden of pulmonary tuberculosis and HIV co-infection in sub-Saharan Africa, it is not surprising that we found pulmonary NTM infection mostly in the 33–44 year-age group. As the ATS/ISDA compliant studies did not describe the clinical characteristics of individual NTM patients, a risk-factor analysis for NTM disease could not be conducted in the present review.

Our review has a number of limitations: we only searched for English language-articles. Given the numbers of Francophone countries in sub-Saharan Africa, French-language publications may have been missed. In addition, our assessment of the clinical relevance of isolated NTM was not as comprehensive as desired because the majority of the studies did not collect the detailed clinical, radiological and microbiological data required to do this. We also could not report the full diversity of NTM in colonization and disease because almost 30% of all isolates were not fully identified to species level. Since the studies reviewed came from varied time periods during which laboratory procedures for ascertainment differed, we cannot exclude the possibility of laboratory procedures before and/or after year 2000 selecting for particular NTM species whilst inhibiting others^64^. For example, the wider usage of sensitive liquid culture media could in theory have selected for specific NTM species. Similarly, the increasing use of molecular methods for identification of current and historical isolates, especially for the MAC and rapidly growing mycobacteria groups, could underpin the changes to NTM taxonomy over time^65–67^. However, we think our results were not significantly affected because the distribution of NTM species identified in the periods before and after 2010 were similar. Given the heterogeneity of studies included in this review including laboratory methods and quality standards, some of the NTM reported here may be due to contamination especially for NTM like *M*.*flavescens* that are frequent laboratory contaminants. It is possible for example that all seven *M*. *flavescens* are contaminants. In more than half of 26 studies that used molecular techniques to identify NTM, 16s rDNA sequencing was used. However, this method has a limitation in that it is not fully capable of distinguishing between all the different NTM species for example *M*. *abscessus* and *M*. *chelonae*. Therefore, it is possible some species have been misidentified or misclassified in these studies.

To conclude, we have provided the first detailed review of pulmonary NTM in sub-Saharan Africa and highlight the contribution of NTM to the aetiology of tuberculous-like pulmonary disease in the sub-continent. Our review also suggests that the presence of NTM as commensals in pulmonary samples may confound the diagnosis of pulmonary tuberculosis, especially in those with a previous history of tuberculosis and/or other chronic respiratory conditions.

Additional research and surveillance is required for investigation of the full contribution of NTM to pulmonary disease, to describe the full repertoire of prevalent and incident NTM, and to determine the role of risk factors (particularly HIV/AIDS) for colonization and/or disease. Given the risk of over diagnosis of NTM in pulmonary samples as tuberculosis disease, resulting in repeated courses of treatment in previously treated tuberculosis patients, investments in, and development of, point of care diagnostics for NTM are required.

### Evidence before this Study

We searched PubMEd, Embase and other databases for the terms “nontuberculous mycobacteria*”, “pulmonary*”, “africa south of the sahara*”, “lung”, and “human”. We searched for English-language articles published up to Oct 1, 2016 and reviewed all eligible articles and their reference lists. Earlier reviews only included NTM isolates, subject level data from just one sub-Saharan Africa country and did not investigate the clinical relevance of isolated NTM.

### Added Value of this Study

This is the first review to utilise all available data to provide a detailed picture of the clinical and molecular epidemiology of NTM isolated from pulmonary samples in sub-Saharan Africa. As a result, we find there is a substantial burden of pulmonary NTM in the sub-continent. With seven out of every 100 presumptive tuberculosis cases either colonised or diagnosed with confirmed pulmonary NTM, the likelihood of pulmonary tuberculosis over diagnosis especially in those with previous history of tuberculosis requires further investigation. In addition, we highlight the knowledge gap resulting from incomplete identification of NTM species.
