# Publisher Correction: Non-tuberculous Mycobacteria isolated from Pulmonary samples in sub-Saharan Africa - A Systematic Review and Meta Analyses

**DOI:** 10.1038/s41598-018-25256-4

**Published:** 2018-05-14

**Authors:** Catherine Okoi, Suzanne T. B. Anderson, Martin Antonio, Sarah N. Mulwa, Florian Gehre, Ifedayo M. O. Adetifa

**Affiliations:** 1 Vaccines and Immunity Theme, Medical Research Council Unit, Fajara, The Gambia; 2 Clinical Services Department, Medical Research Council Unit, Fajara, The Gambia; 3 Microbiology and Infection Unit, Warwick Medical School, University of Warwick, Coventry, United Kingdom; 4 Faculty of Infectious and Tropical Diseases, London School of Hygiene & Tropical Medicine, London, United Kingdom; 5 Disease Control and Elimination Theme, Medical Research Council Unit The Gambia, Fajara, The Gambia; 6 Institute of Tropical Medicine, Antwerp, Belgium; 7 Department of Infectious Diseases Epidemiology, London School of Hygiene and Tropical Medicine, London, United Kingdom; 8 Epidemiology and Demography Department, KEMRI-Wellcome Trust Research Programme, Kilifi, Kenya; 9 College of Medicine University of Lagos, Lagos, Nigeria

Correction to: *Scientific Reports* 10.1038/s41598-017-12175-z, published online 20 September 2017

In the original version of this Article, the authors Ifedayo M.O. Adetifa and Suzanne T.B Anderson were incorrectly indexed. This error has now been corrected.

